# Impact of vitamin D receptor gene polymorphisms (TaqI and BsmI) on the incidence and severity of coronary artery disease: a report from southern Iran

**DOI:** 10.1186/s12872-023-03155-5

**Published:** 2023-03-07

**Authors:** Boshra Akhlaghi, Negar Firouzabadi, Farzaneh Foroughinia, Marzieh Nikparvar, Pouyan Dehghani

**Affiliations:** 1 Department of Clinical Pharmacy, School of Pharmacy, Shiraz University of Medical Sciences, Karafarin Street, PO Box 7146864685, Shiraz, Iran; 2 Department of Pharmacology and Toxicology, School of Pharmacy, Shiraz University of Medical Sciences, Shiraz, Iran; 3 Cardiovascular Research Center, Hormozgan University of Medical Sciences, Bandar Abbas, Iran; 4 Cardiovascular Research Center, Shiraz University of Medical Sciences, Shiraz, Iran

**Keywords:** Coronary artery disease, Vitamin D receptor, Gene polymorphism, TaqI, BsmI, Percutaneous coronary intervention

## Abstract

**Background:**

The association of vitamin D level and vitamin D receptor (VDR) gene polymorphisms with the prevalence of coronary artery disease (CAD) has been evaluated in various studies; however, the reported results were inconsistent. Hence, we aimed to investigate the impact of two VDR gene polymorphisms, TaqI (rs731236) and BsmI (rs1544410), on the incidence and severity of CAD in Iranian population.

**Methods:**

Blood samples were collected from 118 CAD patients underwent elective percutaneous coronary intervention (PCI) and 52 control subjects. Polymerase chain reaction-restriction fragment length polymorphism (PCR-RFLP) was performed for genotyping. SYTNAX score (SS) was calculated as a grading tool for complexity of CAD by an interventional cardiologist.

**Results:**

TaqI polymorphism of VDR was not associated with the incidence of CAD. A significant difference was observed between CAD patients and controls regarding BsmI polymorphism of VDR (p < 0.001). GA and AA genotypes was significantly associated with a decreased risk of CAD (p = 0.01, p-adjusted = 0.01 and p < 0.001, p-adjusted = 0.001 respectively). A allele of BsmI polymorphism was shown to have a protective effect against CAD (p < 0.001, p-adjusted = 0.002). No association was found between TaqI and BsmI polymorphisms of VDR and SS as a measure of CAD severity.

**Conclusion:**

Association of BsmI genotypes with the incidence of CAD revealed that the genetic variation of VDR might play a role in the pathogenesis of CAD.

## Background

Cardiovascular disorders are one of the most important causes of mortality with the prevalence of about 48% among people over the age of 20 [1]. Death from cardiovascular disease (CVD) in 2015 was estimated to be 17.9 million people, of which 7.3 million were caused by coronary artery disease (CAD) [2]. CAD is the most common CVD identified as one of the leading causes of death worldwide. The rising prevalence of CAD in both developed and developing countries imposes a heavy financial burden on these countries [3]. In general, coronary artery occlusion followed by impaired oxygen delivery to the heart muscle leads to angina pain in CAD patients [4, 5]. The major risk factors for CAD include diabetes, high blood pressure, obesity, dyslipidemia, smoking, alcohol consumption, inflammation, diet, and lack of physical activity. Furthermore, vitamin D deficiency can also play a substantial role as a risk factor for CAD [6–8].

Vitamin D is a regulatory hormone that plays an important role in various biological processes including calcium and phosphorus regulation, bone metabolism, and immune and anti-inflammatory responses [9]. Evidence from various studies over the past decade indicates that vitamin D deficiency is associated with an increased risk of CVD, including CAD [10, 11]. Vitamin D exerts its protective cardiovascular effects through various mechanisms. One of these mechanisms is its inhibitory effect on renin biosynthesis, which is involved in the pathogenesis of hypertension. Vitamin D can also have a protective effect against atherosclerosis development [12].

Along the conventional risk factors that predict approximately 50% of the hazards of cardiovascular events [13, 14]; genetics plays the remaining part [15]. To date, different studies have been conducted to recognize numerous genetic variants involved in CAD [16–18]; among which, vitamin D receptor (VDR) gene has been identified as a possible contributor to CVD [19]. So far, a large number of single nucleotide polymorphisms (SNPs) of the VDR have been identified; among which TaqI (rs731236), FokI (rs2228570), BsmI (rs1544410), and ApaI (rs7975232) have been investigated meticulously for their effects on various diseases such as CAD [20].

The SYNTAX score (SS) (synergy between percutaneous coronary intervention with taxus and cardiac surgery) is an angiographic tool to grade the extent and the complexity of lesions in CAD. It helps interventionists to decide the optimal strategy for revascularization. Moreover, SS is a powerful stratification system that provides the possibility for homogenous evaluation of CAD severity [21]. A higher SS demonstrates a more severe coronary involvement and poorer prognosis following coronary intervention [22].

Although there are some papers about the association of VDR gene polymorphisms with CAD, further studies on various ethnic groups needs to investigate this association. This study aimed to investigate the association between two VDR gene polymorphisms (Bsm I and Taq I) and the incidence and severity of CAD in Iranian population, along with studying the association between these SNPs and SS as a powerful tool in stratification of CAD severity for the first time.

## Materials and methods

The study protocol was reviewed and approved by the Ethics Committee of Shiraz University of Medical Sciences (SUMS, Iran) (No: IR.SUMS.REC.1399.1316) and was in accordance with ethical principles of the World Medical Association (Helsinki Declaration). All participants signed the written informed consent prior to participating into the study.

### Subjects

One-hundred and eighteen patients who were admitted to a tertiary cardiac care hospital of SUMS (Ghalb-Al-Zahra hospital) with the diagnosis of CAD were recruited into the study. fifty-two subjects with normal coronary angiography or non-significant CAD (< 50% coronary stenosis) who visited a tertiary care clinic for more CAD evaluation were enrolled in the study as the control group. All of the protocols conformed to the ethical guidelines of the Helsinki Declaration.

Inclusion criteria for the CAD group were as follows: Ages between 18 and 80 years old, confirmed diagnosis of CAD (> 50% luminal stenosis in at least one major coronary artery in angiography) and successful percutaneous coronary intervention (PCI). Exclusion criteria for both groups include severe liver disease, active malignancies, chronic inflammatory disease, history of surgery, or severe trauma in the last month, and administration of immunosuppressive drugs.

All demographic and clinical data were obtained from patients’ histories and medical records. Participants who actively smoked cigarettes were considered as smokers. No documented history of CAD duration before hospitalization was available.

### Coronary angiography

The procedure was performed in a cardiac catheterization laboratory (Cath lab). After local anesthesia, a catheter was guided into the coronary arteries through the femoral or radial artery. Patients received aspirin, clopidogrel, and heparin according to standard protocols prior to coronary angioplasty [23]. All angiographic variables were assessed by an experienced interventional cardiologist who was blinded to other data. SYNTAX score was calculated using the calculator provided by the SYNTAX score website [24]. Patients were divided into two groups based on the SYNTAX score: low risk (SS ≤ 15) and intermediate/ high risk (SS > 15).

### Biological samples and genotyping

Genomic DNAs were extracted from whole blood using DNA extraction kit (Yekta tajhiz, Iran). To identify VDR gene polymorphisms (TaqI and BsmI), polymerase chain reaction-restriction fragment length polymorphism (PCR-RFLP) was performed using specific primers listed in Table 1 [25, 26].

**Table 1:** List of Polymorphisms, primers and digested fragments

Polymorphism	Primers	PCR product (enzyme digestion product)
TaqI	(Forward) 5’-CAGAGCATGGACAGGGAGCAA-3’ (Reverse) 3’-GCAACTCCTCATGGCTGAGGTCTC-5’	740 bp (T: 495 bp + 245 bp; C: 290 bp + 245 bp + 205 bp)
BsmI	(Forward) 5’-CAACCAAGACTACAAGTACGCGTCAGTGA-3’ (Reverse) 5’-AACCAGCGGGAAGAGGTCAAGGG-3’	825 bp (G: 825 bp; A: 650 bp + 175 bp)

PCR protocol for TaqI genotyping was as follows: initial denaturation at 94 °C for 4 min, followed by 35 cycles of denaturation at 94 °C for 50 s, annealing at 66 °C for 50 s, elongation at 72 °C for 50 s, and a final extension at 72 °C for 7 min. PCR conditions for BsmI was similar to TaqI except for the annealing temperature, which was adjusted at 60 °C. The total volume of PCR reaction mixture was 20 µl containing 3 µl nuclease-free water, 1 µl of each reverse and forward primer, 5 µl genomic DNA, and 10 µl PCR Master Mix (Ampliqon).

**Fig. 1:**
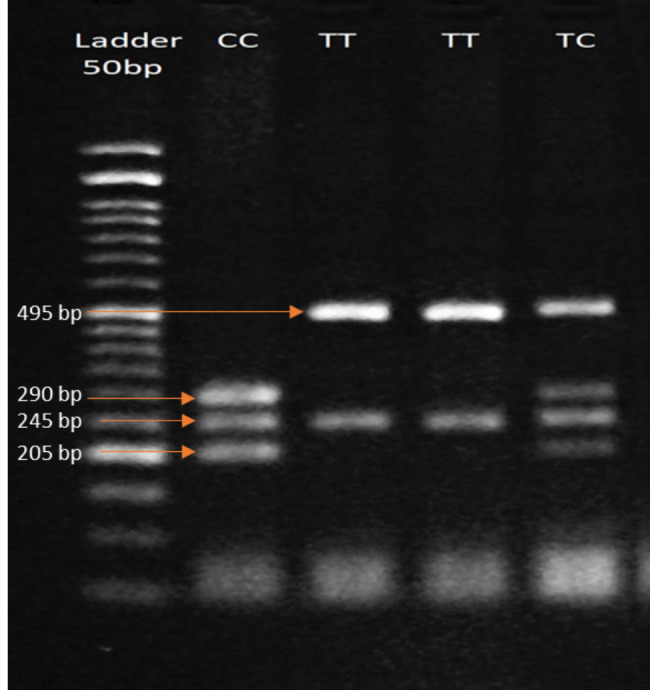
Agarose gel electrophoresis of the PCR–RFLP products digested with TaqI restriction enzyme.TT wild (495 bp, 245 bp), TC heterozygote (495 bp, 290 bp, 245 bp, 205 bp), CC homozygote (290 bp, 245 bp, 205 bp)

**Fig. 2:**
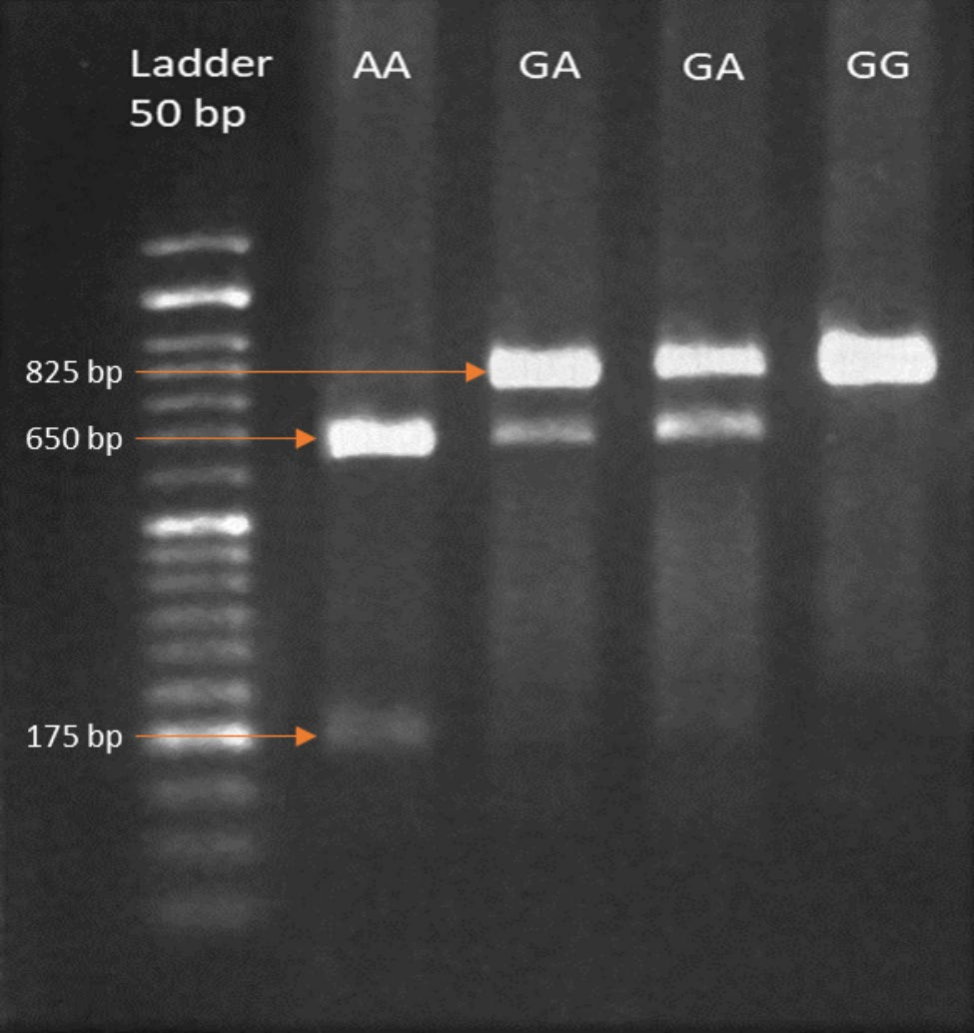
Agarose gel electrophoresis of the PCR–RFLP products digested with BsmI restriction enzyme. GG wild (825 bp), GA heterozygote (825 bp, 650 bp, 175 bp), AA homozygote (650 bp, 175 bp)

After amplification, 18 µl nuclease-free water, 2 µl 10x buffer TaqI, and 1 µl TaqI restriction enzyme (Thermo Scientific) were added to 10 µl of PCR product of TaqI and incubated at 65 °C for 2 h. Two µl of 10x buffer R, and 0.5 µl of BsmI restriction enzyme (Thermo Scientific) were added to 10 µl of PCR product of BsmI and incubated at 37 °C for 3 h. Following enzyme digestion, products were electrophoresed on 2.5% agarose gel at 100 V for 35 min and visualized using a UV transilluminator (Figs. 1 and 2).

### Study endpoints

The primary endpoint of this study was investigation the association between Bsm I and Taq I VDR gene polymorphisms and the incidence and severity of CAD. The incidence of Major adverse cardiac Events over a period of 30 days after coronary angioplasty (30-day MACE) was reported as a secondary endpoint. MACE is defined as myocardial infarction, revascularization treatment, and all-cause death.

According to our previous study [26], there was no association between vitamin D levels and VDR polymorphisms. Therefore; this relationship was not investigated as an endpoint of this study.

### Statistical analysis

Data analysis was carried out using SPSS software version 21 (SPSS Inc, Chicago, USA). All continuous variables were reported as mean ± SD and categorical variables were reported as absolute numbers and percentages. Continuous variables were assessed for normal distribution by the Kolmogorov–Smirnov test and were compared using t-test or Mann–Whitney U test as appropriate. Categorical variables were compared with Chi-square or Fisher exact test as appropriate. Variables with p-value less than 0.2 in the univariate analysis were entered in logistic regression analysis model. Crude and adjusted odds ratios (ORs) were reported with 95% confidence intervals (95%CI). Multiple logistic regression analysis was applied to determine the association between the incidence of CAD and VDR gene polymorphisms and to adjust for the cardiovascular risk factors. P-value < 0.05 was considered significant.

## Results

**Fig. 3:**
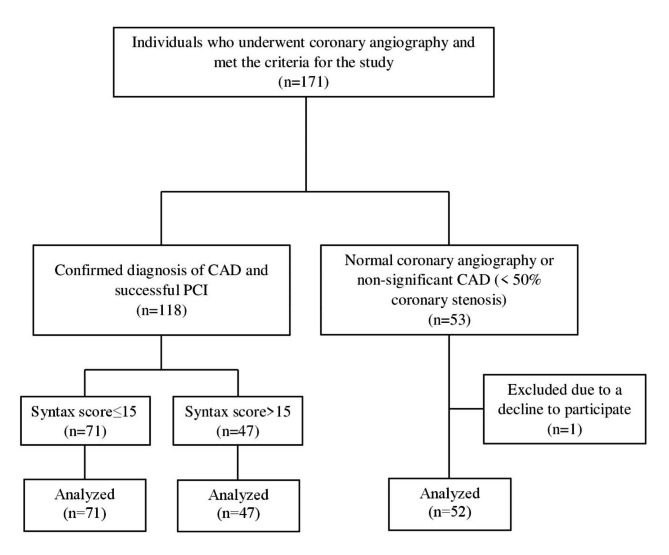
Patient flow diagram

Totally, One-hundred and seventy-one individuals participated in this study. Patient flow diagram is presented in Fig. 3.

Demographic data and clinical characteristics of controls and CAD patients are described in Table 2. There were significant differences between the two groups in terms of age, BMI, sex, diabetes mellitus, hypertension, dyslipidemia, and smoking (all p < 0.05). The mean number of arteries with stents implanted was one vessel for 73.6%, two vessels for 21.8%, and three vessels for 4.5% of patients.

No cases of MACE were found in both groups during 30-day follow up, therefore; evaluation of the impact of VDR gene polymorphisms and patient’s outcomes is not possible.

**Table 2:** The demographic data and clinical characteristics of controls and CAD patients in univariate analysis

Variables	Control (N = 52)	Patient (N = 118)	P-value
Age, years, mean ± SD	51.58 ± 10.48	57.62 ± 10.54	0.001
BMI, kg/m2, mean ± SD	25.20 ± 4.76	27.06 ± 3.94	0.01
Sex			
Male, N (%)	27 (51.90)	80 (67.80)	0.04
Female, N (%)	25 (48.10)	38 (32.20)	
Diabetes Mellitus, N (%)	6 (11.50)	34 (28.80)	0.01
Hypertension, N (%)	16 (30.80)	63 (53.40)	0.006
Dyslipidemia, N (%)	13 (25)	48 (40.70)	0.04
Smoker, N (%)	8 (15.40)	48 (40.70)	0.001
Family history of CAD, N (%)	21 (40.40)	43 (36.40)	0.62

Genotypes and allele frequencies of TaqI and BsmI gene polymorphisms of CAD patients and controls are shown in Table 3. No significant differences were observed neither in genotypes nor in allele frequencies of the TaqI gene polymorphism between the two groups, whereas a significant difference was observed between CAD patients and controls in both genotypes and allele frequencies of the BsmI polymorphism (p < 0.001, OR = 0.14, 95%CI = 0.05–0.35 and p < 0.001, OR = 0.31, 95%CI = 0.19–0.50 respectively). BsmI GG genotype and G allele were more frequent in CAD patients while BsmI AA genotype and A allele were frequently higher in the control group.

**Table 3:** Associations between CAD the incidence and VDR gene polymorphisms (TaqI and BsmI)

Genotypes and alleles		Control (N = 52)	Patient (N = 118)	P-value
**TaqI** Genotype, N (%)	TT	28 (53.80)	59 (50)	
	TC	21 (40.40)	45 (38.10)	0.47
	CC	3 (5.80)	14 (11.90)	
Allele, N (%)	T	77 (74)	163 (69.10)	
	C	27 (26)	73 (30.90)	0.35
**BsmI** Genotype, N (%)	GG	10 (19.20)	58 (49.20)	
	GA	21 (40.40)	43 (36.40)	< 0.001
	AA	21 (40.40)	17 (14.40)	
Allele, N (%)	G	41 (39.40)	159 (67.40)	
	A	63 (60.60)	77 (32.60)	< 0.001

Demographic data and clinical characteristics of CAD patients based on SYNTAX score are described in Table 4. As demonstrated, no significant differences were observed between the two groups (all p > 0.05).

**Table 4:** Demographic data and clinical characteristics of CAD patients based on syntax score in univariate analysis

Variables	Syntax score ≤ 15 (N = 71)	Syntax score > 15 (N = 47)	P-value
Age, years, mean ± SD	56.57 ± 10.43	59.20 ± 10.61	0.19
BMI, kg/m2, mean ± SD	27.14 ± 3.94	26.92 ± 3.97	0.78
Sex			
Male, N (%)	47 (66.2)	33 (70.2)	0.64
Female, N (%)	24 (33.8)	14 (29.8)	
Diabetes Mellitus, N (%)	20 (28.2)	14 (29.8)	0.84
Hypertension, N (%)	38 (53.5)	25 (53.2)	0.97
Dyslipidaemia, N (%)	30 (42.3)	41 (57.7)	0.66
Smoker, N (%)	27 (38.0)	21 (44.7%)	0.47
Family history of CAD, N (%)	29 (40.8)	14 (29.8)	0.22

No association was found between SS and TaqI genotypes and alleles. There was a significant association between SS and BsmI alleles (p = 0.002, OR = 2.29, 95%CI = 1.36–3.85) but not with genotypes (Table 5). The frequency of G allele was higher in CAD patients with SS ≤ 15, while A allele was more frequent in patients with SS > 15.

**Table 5:** The association between SYNTAX score and VDR gene polymorphisms (TaqI and BsmI) in CAD patients

Genotypes and alleles		Syntax score ≤ 15 (N = 71)	Syntax score > 15 (N = 47)	P-value
**TaqI** Genotype, N (%)	TT	32 (45.10)	27 (57.40)	0.38
	TC	29 (40.80)	16 (34)	
	CC	10 (14.10)	4 (8.50)	
Allele, N (%)	T	93 (65.50)	70 (74.50)	0.14
	C	49 (34.50)	24 (25.50)	
**BsmI** Genotype, N (%)	GG	39 (54.90)	19 (40.40)	0.30
	GA	23 (32.40)	20 (42.60)	
	AA	9 (12.70)	17 (14.40)	
Allele, N (%)	G	101 (71.10)	58 (51.80)	0.002
	A	41 (28.90)	54 (48.20)	

Table 6 represents cardiovascular risk factors and VDR gene polymorphisms associated with the incidence of CAD. BsmI GA and AA genotypes were significantly associated with a decreased risk of CAD with adjustment for conventional risk factors (p_a_=0.01, OR = 0.30, 95% CI = 0.11–0.81 and p_a_=0.001, OR = 0.16, 95% CI = 0.05–0.48 respectively). According to the results represented in Table 7, after adjustment for cardiovascular risk factors, A allele demonstrated a protective effect against CAD development (p_a_=0.002, OR = 0.23, 95%CI = 0.09–0.59).

**Table 6:** The association between the incidence of CAD and cardiovascular risk factors and BsmI genotypes

Variables	P-value	Crude OR (95% CI)	P_a_-value	Adjusted OR (95% CI)
Sex (male)	0.04	1.94 (1.00–3.79)	0.04	2.60 (1.00-6.75)
Age	0.001	1.05 (1.02–1.09)	0.01	1.05 (1.01–1.09)
BMI	0.01	1.11 (1.02–1.21)	0.04	1.11 (1.00-1.23)
Diabetes Mellitus	0.01	3.10 (1.21–7.93)	0.05	2.98 (0.97–9.20)
Hypertension	0.007	2.57 (1.29–5.14)	0.12	2.15 (0.81–5.70)
Dyslipidemia	0.05	2.05 (0.99–4.25)	0.34	1.59 (0.60–4.21)
Smoking	0.002	3.77 (1.63–8.71)	0.004	4.72 (1.62–13.79)
BsmI genotype				
GG	reference 0.01		reference 0.01	
GA	0.01	0.35 (0.15–0.82)	0.01	0.30 (0.11–0.81)
AA	< 0.001	0.14 (0.05–0.35)	0.001	0.16 (0.05–0.48)

P_a_: adjusted p-value; OR: Odds Ratio; CI: Confidence Interval

**Table 7:** The association between the incidence of CAD and cardiovascular risk factors and BsmI alleles

variables	P-value	Crude odds ratio (95%CI)	P_a_-value	Adjusted odds ratio (95% CI)
Sex (male)	0.04	1.94 (1.00–3.79)	0.04	2.66 (1.03–6.87)
Age	0.001	1.05 (1.02–1.09)	0.006	1.05 (1.01–1.10)
BMI	0.01	1.11 (1.02–1.21)	0.03	1.11 (1.01–1.23)
Diabetes Mellitus	0.01	3.10 (1.21–7.93)	0.05	3.04 (0.99–9.35)
Hypertension	0.007	2.57 (1.29–5.14)	0.13	2.07 (0.78–5.44)
Dyslipidemia	0.05	2.05 (0.99–4.25)	0.38	1.53 (0.58-4.00)
Smoking	0.002	3.77 (1.63–8.71)	0.003	4.90 (1.69–14.25)
BsmI allele				
G	reference		reference	
A	< 0.001	0.24 (0.11–0.53)	0.002	0.23 (0.09–0.59)

P_a_: adjusted p-value; OR: Odds Ratio; CI: Confidence Interval

Moreover, the power analysis was estimated 98% to detect an effect size of 0.7893 using 5 degrees of freedom chi-square test with a significance level (α) of 0.05 for BsmI genotypes in patients and control group.

As demonstrated in Table 8, no significant association was found between SS and BsmI alleles after logistic regression analyses.

**Table 8:** The association between SYNTAX score and cardiovascular risk factors and VDR gene polymorphisms

variables	P-value	Crude OR (95%CI)	P_a_-value	Adjusted OR (95% CI)
Age	0.19	1.02 (0.98–1.06)	0.11	1.03 (0.99–01.07)
BsmI allele				
G	reference		reference	
A	0.12	1.79 (0.85–3.79)	0.06	2.09 (0.94–4.63)

P_a_: adjusted p-value; OR: Odds Ratio; CI: Confidence Interval

## Discussion

The present study was conducted to investigate the association between TaqI and BsmI polymorphisms and the incidence and severity of CAD in Iranian population. This is the first study in which the association of TaqI and BsmI polymorphisms of VDR and SS has been evaluated in CAD patients. Based on our findings, no significant association was found between SS and BsmI polymorphism. However, a significant association was found between BsmI genotypes and alleles and the incidence of CAD. GA and AA genotype carriers showed a lower risk of developing CAD and the A allele was found to have a protective effect against CAD.

Association between vitamin D deficiency and the prevalence of CVD risk has been investigated in various studies [27–30]. Evidence from Framingham Offspring Study demonstrated a higher rate of serious cardiovascular events (up to 80%) in participants who were vitamin D deficient [31]. Also, a cohort study carried out in India indicated a normal level of vitamin D in less than 5% of patients with CAD. Concordantly, it was suggested that vitamin D deficiency is very common in CAD [32]. It is shown that a low level of vitamin D is associated with a rise in blood pressure and cardiovascular risk [6]. Vitamin D may play a role in the development of atherosclerosis through affecting calcification or various pathways influential in the process of inflammation [33]. A study on Iranian patients under coronary computed tomography angiography (CCTA) showed that there was a correlation between vitamin D deficiency and coronary artery calcification as well as stenosis severity [34]. Results of a study showed that a low level of vitamin D was associated with structural changes in the heart including systolic and diastolic dysfunction [35]. Another study demonstrated that the levels of matrix metalloproteinase-9 (MMP-9), a contributor to atherosclerosis process and heart remodelling, were higher in CAD patients with lower vitamin D levels [36]. In addition, vitamin D suppresses the expression of the renin gene and down-regulates the renin-angiotensin system (RAS) which is a major contributor to hypertension and cardiac remodelling [37].

It is reported in several studies that vitamin D exerts its physiological effects through VDR [38]. VDR is the intracellular receptor of vitamin D that binds to the active form of this hormone and exerts various biological effects by interacting with particular nucleotide sequences in targeted genes [39]. Inactivation of VDR results in an increase in RAS activity, endothelial dysfunction, hypertension, and cardiac hypertrophy [40]. The effect of vitamin D supplements on reducing angiotensin II levels, plasma renin activity, and blood pressure has been demonstrated in clinical studies [41, 42]. Among the identified SNPs of VDR, BsmI, TaqI, FokI, and ApaI have gained much attention owing to their possible impact on predicting a variety of pathophysiologic or physiologic phenotypes including CVDs [25]. However, controversies on the association of VDR polymorphisms with susceptibility to CVD have been reported in various studies [43, 44]. Based on our findings, a significant association was found between SS and alleles of BsmI; however, after including the confounding factors using logistic regression models, no association was observed between BsmI alleles and severity of CAD. Moreover, a significant association was found between VDR BsmI genotypes as well as associated alleles and the incidence of CAD. After evaluating the effects of confounding factors by multivariate logistic regression analysis, it was found that GA and AA genotype carriers have a lower risk of developing CAD (p = 0.01, OR = 0.30 and p = 0.001, OR = 0.16 respectively) and the A allele was found to have a protective effect against CAD (p = 0.002, OR = 0.23). In line with our findings, Ortlepp et al. concluded that carriers of GG genotype were at higher risk of CAD and type 2 diabetes [45]. In addition to these studies, a meta-analysis study demonstrated that carriers of the AA genotype were at a lower risk of hypertension in comparison to those carrying the GG or GA genotypes concluding that AA genotype plays a protective role against CVD [46]. Eweida et al. and Raljević et al. reported that GA genotype was less frequent in CAD patients compared to healthy individuals, whereas AA genotype was more frequent in CAD patients [27, 47]. The authors suggested that carriers of AA genotype are more susceptible to CAD while GA genotype plays a protective role [47]. Moreover, the G allele was assumed to have a protective effect against CVD, and the A allele was reported as a potential predictor of CVD risk, which is in contrast with our findings [27]. In line with these studies, a study on a population from west of Iran proposed the A allele as a possible predictor for CAD development [48]. On the other hand, some studies and meta-analyses did not find any significant association between BsmI polymorphism and the incidence or severity of CAD [20, 33, 44]. Discrepancies between results of these studies can be ascribed to different ethnicities of the study populations, different sample sizes, and heterogeneity of CAD severity and CAD definition.

Results of our study revealed that TaqI polymorphism was not associated with the incidence and severity of CAD. This finding is supported by several other studies. He et al. reported no association between TaqI polymorphism and risk of CAD [49]. Similar result was observed in Egyptian males [50]. A meta-analysis conducted in an Iranian population also revealed no significant association between TaqI polymorphism and CAD [20]. In contrast, results of a French study reported that the C allele (minor allele) of TaqI polymorphism was associated to an increased risk of CAD in patients with type 2 diabetes [51]. Moreover, the results of a study demonstrated that C allele and TC genotype significantly predict the risk of CAD development [52]. In another study, CC genotype was more prominent among the CAD patients who had experienced myocardial infarction [47].

It is not clear how VDR gene polymorphisms play a role in the pathogenesis of CAD. It is thought that vitamin D binding sites may be altered by some VDR gene polymorphisms including TaqI polymorphism, which affects the function of VDR and may lead to inflammatory responses participating in an increased risk of developing atherosclerosis and CAD [52]. The BsmI polymorphism which is located in intron 8 near the 3′ end of the VDR gene, does not change the VDR protein amino acid sequence. Nevertheless, it can alter mRNA stability, disrupt mRNA transcription splice sites, or change intronic regulatory elements which may lead to alteration of gene expression. The TaqI polymorphism in exon 9 does not alter the VDR protein but it plays a role in the regulation of mRNA stability [53].

Our study has some strengths as well as limitations. It is noteworthy that using SS, as a powerful stratification system, in order to provide a homogenous evaluation of CAD severity and recruiting studied groups from an ethnically homogenous population were the strengths of this study. One of the limitation of this study was the small sample size of enrolled participants which was attributed to the limited number of participants with normal coronary angiography.

Considering the controversial results with respect to the association of VDR gene polymorphisms with the incidence and severity of CAD, further studies on various ethnic groups with larger sample sizes are urged. The mechanistic pathway by which these polymorphisms affect CAD also needs to be investigated in future pharmacological studies.

## Conclusion

Association of BsmI genotypes with the incidence of CAD revealed that along with vitamin D level, the genetic variation of its receptors might also play a role in the pathogenesis of CAD and may propose this variant as a marker of risk assessment for CAD. Therefore, assessment of BsmI polymorphism may be considered as a new approach for the assessment of CAD risk and may be a helpful measure in designing a better clinical approach for the prevention and management of CAD patients.

## Data Availability

The datasets used and/or analysed during the current study are available from the corresponding author on reasonable request.

## Abbreviations

CVD: Cardiovascular disease
CAD: Coronary artery disease
VDR: Vitamin D receptor
SNPs: Single nucleotide polymorphisms
SS: SYNTAX score
PCI: Percutaneous coronary intervention
PCR-RFLP: Polymerase chain reaction-restriction fragment length polymorphism
CCTA: Coronary computed tomography angiography
MMP-9: Matrix metalloproteinase-9
